# Effectiveness of dissemination strategies of maternal clinical guidelines: A narrative review

**DOI:** 10.4102/phcfm.v16i1.4494

**Published:** 2024-12-17

**Authors:** Eunice N. Atsali, Doreen Kaura, Mark Tomlinson

**Affiliations:** 1 Department of Nursing and Midwifery, Faculty of Medicine and Health Sciences, Stellenbosch University, Cape Town, South Africa; 2 Institute for Life Course Health Research, Stellenbosch University, Cape Town, South Africa

**Keywords:** clinical guidelines, dissemination strategies, maternal care, primary health care, skilled birth attendants

## Abstract

**Background:**

Maternal clinical guidelines (MCGs) provide evidence-based recommendations for skilled birth professionals (SBPs) at the point of care. The dissemination strategies and use of MCGs are inconsistent among skilled birth providers despite their potential to improve the maternal care outcomes.

**Aim:**

This study examined the effectiveness of dissemination strategies of MCGs by SBPs in a primary care setting.

**Method:**

We searched in Medline, PubMed, CINAHL and Google Scholar. Search terms were effectiveness, dissemination and use, MCGs, SBPs and primary health care facilities. Studies published in English, conducted between 2010 and 2023 and focussing on dissemination strategies and use of MCGs were included. The final articles were presented in narrative format.

**Results:**

The search yielded 212 studies. After removing duplicates, seven articles that met the inclusion criteria for the review were included. The narrative review summarised the findings as: Use of MCGs which showed the barriers and enablers for the use of maternal guidelines. The level of adherence to MCGs was summarised, and one study showed the use of support supervision and collaboration improved aspects of MCGs.

**Conclusion:**

The findings show how skilled attendants acknowledge that MCGs can contribute to improving maternal outcomes. They further describe how, in practice, they are rarely used at the facility level. There is a need for more research on dissemination strategies to ensure improved use of MCGs in primary health care facilities.

**Contribution:**

We highlight the key gap in the dissemination of MCGs at primary health care facilities which if improved can potentially improve the use of MCGs.

## Introduction

Globally, birth-related complications account for more than 287 000 maternal deaths each year, primarily because of preventable causes,^1^ with most occurring in low- and middle-income countries.^2,3,4^ Several strategies and interventions have been put in place to avert maternal mortality as well as improve the quality of care for women.^1,2,3,4,5^ One critical strategy for effective quality interventions in practice is the use of evidence-based guidelines.^5,6,7^

The World Health Organization (WHO) together with other international organisations such as the United Nations Population Fund (UNFPA)^8^ have developed a series of evidence-based guidelines for the management of maternal conditions. These guidelines are routinely adopted, adapted or contextualised in other countries. In Kenya for example, the Ministry of Health (MOH), with its partners, takes the lead in contextualising WHO guidelines to the Kenyan context. They contextualise the Kenyan guidelines and protocols with guidance and support from the WHO^9^ and other health partners.

Maternal clinical guidelines (MCGs) are significant in providing unified evidence-based recommendations for healthcare professionals, especially skilled birth professionals (SBPs), in client care for specific conditions.^10,11,12,13,14^ Globally, it is expected that SBPs attend 90% of all births.^15^ However, in most low- and middle-income countries, SBP maternal care is still trailing at just above 60% compared with developed countries, which are at above 95%.^16,17,18^ This is also lower than the global target of above 90%. Despite efforts to improve hospital births by different countries, such as free maternity care,^19^ it is still a challenge to attain the global target.

There are several challenges in the effective dissemination and use of MCGs. Studies conducted in high-income countries identify awareness, attitude to change and inadequate training,^5,20,21,22,23^ as the major challenges to implementation of guidelines. The situation is similar in sub-Saharan Africa with the challenges being compounded by staff shortage.^24,25,26,27^ In most low- and middle-income counties, there is a severe shortage of skilled birth health professionals.^28^ In some regions there is inequality in the distribution of SBPs between different levels of care and regions within a country. Some regions especially in urban areas have adequate numbers, while rural areas have severe shortages of SBPs.

This severe shortage and resource constraints compromise evidence-based care in healthcare facilities.^26,27^ While there are guidelines to support evidence-based care, especially in maternal health,^28,29^ the dissemination and use seem to be inconsistent in different regions.^24,27^ Consequently, maternal mortality is high in some areas, while it is low in other areas.^24^ Similarly, the dissemination and use of MCGs are inconsistent among SBPs despite their potential to improve the quality outcomes of maternal healthcare provision.^30,31,32^

The Kenyan guidelines have addressed this by highlighting how the guidelines will be disseminated.^9^ However, this has not addressed the effectiveness of the strategies recommended in disseminating and using the MCGs. This review, therefore, examined the effectiveness of dissemination strategies and the use of MCGs by SBPs in primary care settings.

### Participants

The review considered studies that included SBPs working within primary health care settings. We used the WHO definition of an SBP,^33^ which is an educated, licensed and competent practising midwife, nurse or doctor who manages women and neonates across the health continuum.^28^ Skilled birth professionals needed to have at least 1 year of experience. The participants needed to have been involved in guidelines dissemination and use. Studies were excepted if they involved skilled birth attendants who had worked less than 1 year, or were not involved in actual use of guidelines.

### Interventions or strategies

Several different dissemination strategies have been examined in the literature, including interactive workshops, audits and feedback, and distribution of printed materials.^34,35,36^ We examined strategies specific to primary health care contexts.

### Comparator

Usual care without guideline use and dissemination strategy.

### Outcomes

This review considered studies with the following outcomes:

- Successful dissemination strategies of MCGs.
- Adherence to the use of maternal guidelines by SBPs.
- Improved utilisation of maternal guidelines.

### Context

The context of the study is global. World Health Organization defines primary health care as a community-oriented approach that aims at attaining the highest health wellness through prevention, treatment, rehabilitation and promotion in an individual’s environment.^1^

In this study, primary health care facilities are those providing promotive, curative, rehabilitative and palliative maternal healthcare at levels one, two and three. Level one facilities are community and household facilities, level two are facilities providing maternal care based within the community, and level three facilities are health centres which provide comprehensive maternal healthcare.

### Types of sources

This review considered quantitative and mixed methods studies. Quantitative studies included surveys only, as randomised controlled trials focussing on our review were not found. The quantitative results of mixed methods reviews were also included. This was considered because of the paucity of literature during the search.

## Methods

This narrative review was conducted as per the protocol registered through the National Institute for Health Research PROSPERO international prospective register review (21 February 2022; registration number: CRD42022244279). This article is the first in a series of Mixed Methods Systematic Reviews (MMSRs) in line with the Joanna Briggs Institute (JBI) methodology for MMSR.^37^ This article covers the quantitative aspect of the review conducted.

### Search strategy and databases

We consulted a health sciences librarian at Stellenbosch University (SU) to develop the search strategy. A brief initial search of Medline and CINAHL was conducted to inform the broader search. The keywords from the titles and abstracts of articles found were then used to develop the search strategy for Cochrane Review Library, PubMed, CINAHL, Medline, EMBASE, Scopus and Web of Science. The keywords searched were ‘Dissemination and use’, ‘maternal clinical guidelines’, ‘skilled birth professionals’, ‘health facilities, experiences’, ‘skilled’, ‘parturition’, ‘birth’, ‘attendants’, ‘dissemination’, and ‘primary health facilities’. Additional searches were conducted from references of the studies (see Search strategy Appendix 1, TABLE 1-A1). Research articles published between 2010 and July 2023 in the English language were included.

### Study selection

Identified studies were uploaded in the Mendeley citation manager, and duplicates were removed. E.N.A. and D.K. independently reviewed the titles, abstracts and full texts of the studies that met the criteria. In case of disagreements between the reviewers, a third reviewer, M.T., counter-checked and resolved. Studies included in the study must focus on skilled birth attendants. They must be disseminating and implementing guidelines using specific strategies. Maternal guidelines were the focus and adherence to the guidelines was included. Studies that did not meet the criteria were excluded.

### Assessment of methodological quality

A standardised critical appraisal tool was independently used by two reviewers to assess the quality of studies.^38^

All studies, regardless of the results of their methodological quality, underwent data extraction and synthesis. The JBI critical appraisal tool was used to check data quality. The studies were incorporated for review after critical appraisal from E.N.A. and D.K., and an agreement was reached.

Following critical appraisal, studies that did not meet the quality threshold were excluded. Those excluded were based on a score of less than five out of a possible eight points. This decision was based on a list of the rules per JBI Sumari.

### Data extraction

Data were extracted using the standardised JBI tool in JBI SUMARI (see Appendix 2, TABLE 1-A2). The specific components include authors’ names, study aims, participants and setting. It also included the findings from each study. We used a narrative synthesis because of heterogeneous findings. The outcomes and interventions were also diverse; therefore, we analysed the findings as per the search outcome.

### Ethical considerations

Ethical clearance to conduct this study was obtained from the Stellenbosch University Health Research Ethics Committee (HREC) (No. S21/02/024) and Amref Health Africa, Amref Ethics and Scientific Review Committee (ESRC) (No. ESRC P1044/2021).

## Results

**FIGURE 1:**
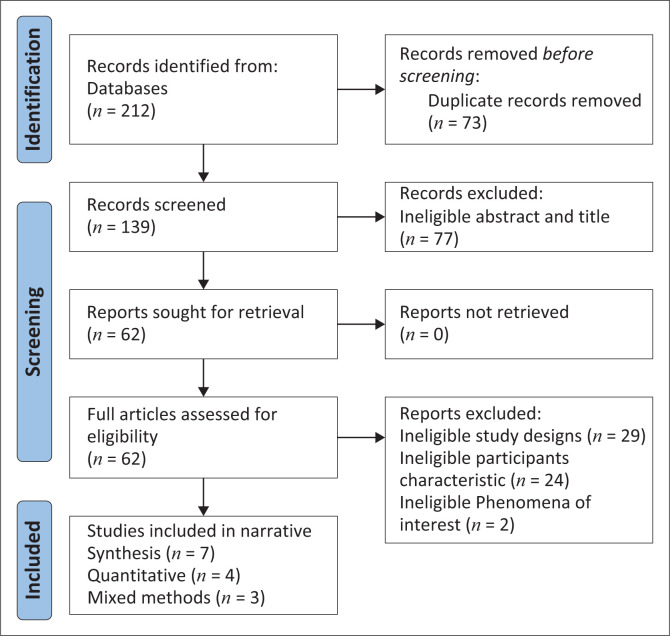
Preferred reporting items for systematic reviews and Meta-Analyses for this study.

The initial search yielded 212 studies, resulting in 139 records after duplicates were removed (see Figure 1). A total of seven studies met the eligibility criteria (four quantitative and three mixed methods studies). Results are summarised in the PRISMA flow chart (see Figure 1).

## Review findings

Seven articles met the criteria for this study. Two of the articles included were from Australia: one focussing on the implementation of alcohol screening guidelines at antenatal care (ANC), and the second focussed on clinical guidelines used to reduce stillbirths at ANC. One article included was from Latin America focussing on post-partum haemorrhage protocol adherence. One article was from England and focussed on guideline implementation on Gestational Diabetes Mellitus Screening at ANC. One article was from Canada focussing on guidelines on screening of obesity during ANC. Two articles were from Uganda: one focussed on postpartum haemorrhage guidelines use, while another focussed on the effect of support supervision on maternal services and practice. Of the seven articles, four were quantitative and three were mixed methods. Quantitative data were extracted from the mixed methods studies. In the narrative synthesis, the results were summarised as: use of MCGs, adherence to MCGs and improved utilisation of MCGs. However, the outcome of successful dissemination strategies of MCGs was not met as the articles focussed on implementation more than dissemination.

### Use of maternal clinical guidelines

Of the studies identified, four articles focussed on factors influencing the use of maternal guidelines, mainly on barriers and enablers for the use of MCGs.

#### Barriers to the use of maternal clinical guidelines

Three articles identified barriers to the use of MCGs.

An article by Doherty^39^ used a theoretical domain framework to examine barriers to the implementation of clinical guidelines for alcohol consumption among women at antenatal services in the United Kingdom (UK). Eight SBPs completed the survey. The main barriers were summarised as 11 domains: knowledge, skills, professional position and identity, beliefs of individual competencies, beliefs about penalties, motivation and goals, memory, attention and decision process, environment and resources, social effects, emotional regulation and behaviour. Among clinicians, mainly doctors, the main barriers identified were environmental context, social influences, beliefs about capabilities and behavioural regulation. Among the managers, who were mainly midwives, the main barriers identified were emotional regulation and environmental context and resources. In this study, the findings indicate that the main barriers to implementing guidelines are related to environment and resources. The results suggest that when guidelines are developed, all barriers must be considered. This will potentially help in developing strategies that cater for all SBPs in healthcare.

A national survey by Bell et al.^22^ on health professionals’ implementation and use of national screening guidelines for gestational diabetes in England found a 40% compliance with National Institute for Healthcare Excellence (NICE) guidelines. The main barriers to the guideline’s compliance were a lack of capacity among the health professionals, and inadequate funding and resources. Some trusts (41%) used different diagnostic criteria for Gestational Diabetis Mellitus (GDM) different from those provided in the NICE guidelines.

Davies-Tuck et al.,^40^ in their cross-sectional survey to understand the views of the staff in changing clinical practice to reduce stillbirth among Asian women, identified several barriers. The staff reported increased workload and concerns around rationale, access to care, resources, a lack of education, client safety and communication issues with non-English speakers.

#### Enablers for use of maternal clinical guidelines

Three articles identified enablers for the use of MCGs.

A cross-sectional survey by Davies-Tuck et al.^40^ was conducted to understand the views of the staff in changing clinical practice to reduce stillbirth among Asian women. It identified that most of the 120 surveyed staff agreed on the need for clear and applicable clinical guidelines.^37^ The main enablers for the use of maternal guidelines in this article were: the staff understanding reasons behind the guidelines (79%), the staff knowing its intended audience (83%) and the staff knowing procedures (74%).

A national survey by Bell et al.,^22^ on health professionals’ implementation and use of national screening guidelines for gestational diabetes in England, found that 81% of trusts offered Oral Glucose Tolerance Test (OGTT) in the second trimester for women at risk of developing gestational diabetes. However, the compliance to NICE guidelines was only 40%. Compliance was based on NICE guidelines and included: a previous history of GDM (25%) and a body mass index (BMI) of over 30 kg/m^2^ (22%). The main enabler identified was the clinician’s knowledge of the baseline BMI in accordance with NICE guidelines.

Murray-Davis and colleagues^41^ showed that the majority of midwives (93%) reported awareness of obesity care protocols. They however had a difference in knowledge of when to transfer care whether below or above a BMI of 45. Most respondents reported that BMI guidelines were not well understood within the midwifery curriculum in colleges. They reported that they understood the guidelines better during clinical practice. The main enablers for the use of the guidelines were continuous professional developments (CPDs) by professional organisations. Collaborative care was considered an enabler for the use of guidelines by half (50%) of the midwives.

### Level of adherence to maternal clinical guidelines

In this review, adherence refers to the commitment of skilled birth attendants to use MCGs. Two studies highlighted the level of adherence to MCGs. Both studies highlighted a low level of adherence to MCGs. However, Braddick and colleagues,^42^ in their study, highlighted that clinicians were more likely to adhere to individual aspects of guidelines unlike when it is a bundle. Braddick and colleagues^42^ assessed the level of adherence to postpartum haemorrhage clinical guideline recommendations in Uganda. The study highlighted high adherence to specific aspects of WHO Post Partum Haemorrahge (PPH) management recommendations. Criteria included Active Management of Third Stage of Labour (AMSTL) the components were administration of uterotonic within 1 min of birth (68.2%), controlled cord traction (77.3%) and delayed cord clamping (61.7%). However, the study further showed low adherence to all three AMTSL guidelines at only 34%. This highlights how important some aspects of AMTSL are considered by SBPs such as controlled cord traction while other aspects such as delayed cord clamping seem to be of low importance.

The study by Olmedo et al.,^43^ on improving maternal health and safety through adherence to postpartum haemorrhage protocol in Latin America, examined health professionals at the national level, regional level and local levels. They found that adherence to AMTSL guidelines at the national level was at 29%, regional level at 3% and local level at 46%. The study examined adherence to three interventions: administration of a uterotonic drug after the birth of the baby, controlled cord traction for placenta birth and uterine massage following placental birth. In all three levels of care, healthcare professionals had low adherence to AMTSL guidelines. The study further highlighted that provider training and the retention of experienced healthcare providers were not associated with greater adherence to protocols. In contrast, at the regional level where all the SBPs had been trained on AMTSL, the adherence was lowest. The observation findings identified provider belief, a lack of oxytocin, and the point of birth as contributing factors to non-adherence. This study indicates that, at the local level, where SBPs had fewer years of experience, adherence was higher. This suggests that previous experience influences adherence to clinical guidelines.

### Improved utilisation of maternal clinical guidelines

Improved use of guidelines in this review refers to the relationship between the use of guidelines before a given dissemination strategy and after the use of the dissemination strategy. One article described factors leading to improved utilisation of MCGs. Kisakye et al.^25^ examined the effects of support supervision on maternal newborn practices in Uganda. Maternal and Neonatal Implementation for Equitable System (MANIFEST) project was implemented on 28 rural health facilities. The project implemented multidisciplinary support supervision coupled with mentorship to SBPs. They supervised different aspects; but for this study, we examined maternal outcomes aspect only. Three supervision supports were implemented on a quarterly basis and an audit was performed. The supervision led to an improvement in the availability of oxytocin from 57% to 82%. Assisted vaginal birth improved from 7% to 21% by the third supervision. Manual removal of products of conception improved from 14% to 54% and vitamin K administration from 21% to 43%. The study indicated that guidelines aspects improved over three supervision visits. The facilities were more likely to improve with supervision and mentorship.

## Implications and recommendations

This review aimed to determine the effectiveness of dissemination strategies and the use of MCGs. This narrative review identified seven studies intended to achieve the outcomes: use of MCGs by SBPs, level of adherence to the use of MCGs and improved utilisation of MCGs. Four of the studies addressed the use guidelines outcomes.^22,40,42^ The findings in the four studies were summarised as enablers and barriers to the use of MCGs. The enablers identified are like those of other studies such as staff understanding the rationale for guidelines^41,44,45^; staff being familiar with the procedures^46^; knowledge of guidelines^47,48^; CPDs during clinical practice^13,49^ and collaborative care.^41,45,50,51^ These findings support the theory of planned behaviour change which highlights the importance of self-drive in enabling a particular practice.^49^ In this study, the enablers were mainly focussed on the SBPs’ perceived ability to utilise the guideline. This was influenced by their confidence in knowing the guidelines and its rationale through CPDs, and collaborative care. This indicates that utilisation of MCGs can be best achieved by influencing individual factors of SBPs which consequently can improve confidence to use MCGS.

The barriers to the use of MCGs identified in this review are similar to those found in earlier studies. They include barriers related to environmental factors,^44,46,52^ limited resources^50,52,53,54^ and lack of capacity among healthcare professionals.^41,55,56^ Other challenges include increased workload, a lack of rationale for guidelines, access issues, insufficient education and concerns about client safety.^21,26,57,58^ While some of the barriers are general healthcare barriers for implementation of MCGs, there is a need to focus on improving systemic factors that prevent the use of MCGs. Behaviour change among SBPs can greatly be improved if they feel in control. This control can be achieved if the systemic barriers are removed. Adherence to MCGs can be improved with emphasis on individual factors, systemic factors and environmental factors.

In this review, the findings indicated low adherence to the use of MCGs, particularly when the implementation of guidelines requires multiple interventions. This aligns with earlier studies that highlight the importance of simplified guidelines in improving their uptake.^44,45,52^ Other studies have shown high adherence to guidelines when the SBPs are skilled and experienced.^47,48^ This review identified a low level of adherence in the more skilled workforce. This can be related to resistance to behavioural change. In the theory of planned behaviour change, a developed norm ensures the completion of a behaviour.^59^ However, when the norm is challenged, the SBPs may resist changing their actions.

One study focussed on improved utilisation of guidelines. However, it focussed on three dissemination strategies for utilisation of MCGs: mentorship, support supervision and collaboration.^43,60^ There is, however,61 a strong suggestion for the use of multiple strategies in implementing MCGs. Similarly, some studies such as a systematic review by Medves et al.^61^ have shown that the use of varied dissemination and implementation strategies improved SBP practice. The study, however, did not conclude which combinations were more effective. There is a need to examine the dissemination and use of strategies that work for MCGs, especially in low- and middle-income countries.

## Strength and limitations

The target of this study was SBPs whose impact in improving maternal outcomes is key. The limitation for this study is that the search did not yield adequate studies that would meet the meta-analysis threshold. While the researchers’ intention was to include randomised control trials, the search did not yield any study focussing on the dissemination of MCGs at primary health care facilities. Moreover, some of the studies included in this review focussed on small populations which might not be generalisable to the whole population. The authors of this study, however, included studies from different continents which had comparable results. Therefore, the findings of this study form a basis for future empirical studies focussing on the effectiveness of dissemination and implementation strategies at primary health care facilities.

## Conclusion

This review examined the effectiveness of dissemination strategies and the use of MCGs by SBPs in the primary care setting. However, after the search, the focus of MCGS at the primary health care facilities was limited. The findings of this study indicate that SBPs acknowledged that MCGs improve maternal outcomes; however, their use is still limited at healthcare facilities. Furthermore, effective dissemination strategies improve the optimum use of the guidelines during maternal care. There is a need to conduct more studies on the dissemination strategies that are effective in the use of maternal guidelines.

## Appendix 1

**TABLE 1-A1:** Search strategy.

Database	URL	Search strategy or terms	No. of hits
Pubmed	https://www.ncbi.nlm.nih.gov/pmc/?term=(experiences%5BAll+Fields%5D+AND+skilled%5BAll+Fields%5D+AND+(%22parturition%22%5BMeSH+Terms%5D+OR+%22parturition%22%5BAll+Fields%5D+OR+%22birth%22%5BAll+Fields%5D)+AND+attendants%5BAll+Fields%5D+AND+dissemination%5BAll+Fields%5D+AND+(%22mothers%22%5BMeSH+Terms%5D+OR+%22mothers%22%5BAll+Fields%5D+OR+%22maternal%22%5BAll+Fields%5D)+AND+clinical%5BAll+Fields%5D+AND+(%22guideline%22%5BAll+Fields%5D+OR+%22guidelines+as+topic%22%5BMeSH+Terms%5D+OR+%22guidelines%22%5BAll+Fields%5D))+AND+(primary%5BAll+Fields%5D+AND+(%22health+facilities%22%5BMeSH+Terms%5D+OR+(%22health%22%5BAll+Fields%5D+AND+%22facilities%22%5BAll+Fields%5D)+OR+%22health+facilities%22%5BAll+Fields%5D))&cmd=DetailsSearch	(experiences[All Fields] AND skilled[All Fields] AND (“parturition”[MeSH Terms] OR “parturition”[All Fields] OR “birth”[All Fields]) AND attendants[All Fields] AND dissemination[All Fields] AND (“mothers”[MeSH Terms] OR “mothers”[All Fields] OR “maternal”[All Fields]) AND clinical[All Fields] AND (“guideline”[All Fields] OR “guidelines as topic”[MeSH Terms] OR “guidelines”[All Fields])) AND (primary[All Fields] AND (“health facilities”[MeSH Terms] OR (“health”[All Fields] AND “facilities”[All Fields]) OR “health facilities”[All Fields]))	124
Medline	https://lwwreprints.ovidds.com/discover/results?q=Dissemination+and+use+AND+maternal+clinical+guidelines+AND+skilled+birth+attendants+AND+health+facilities+&page=3	Dissemination and use AND maternal clinical guidelines AND skilled birth professionals AND health facilities	61
CINAHL	http://web.b.ebscohost.com.ez.sun.ac.za/ehost/resultsadvanced?vid=2&sid=32bbbb6c-b219-454c-b8d8-05012f007e5b%40pdc-v-sessmgr03&bquery=Maternal+clinical+guidelines+AND+Dissemination+AND+Use+OR+Implementation+AND+Skilled+birth+attendants+AND+(+experiences+or+perceptions+or+attitudes+or+views+or+feelings+or+qualitative+or+perspective+)&bdata=JmRiPWNpbjIwJnR5cGU9MSZzZWFyY2hNb2RlPVN0YW5kYXJkJnNpdGU9ZWhvc3QtbGl2ZSZzY29wZT1zaXRl	Maternal clinical guidelines AND Dissemination AND Use OR Implementation AND Skilled birth professionals AND (experiences or perceptions or attitudes or views or feelings or qualitative or perspective)	27

## Appendix 2

### Summary of the findings

#### Quantitative studies

**TABLE 1-A2:** Data extraction instrument.

Author	Findings	Interpretation of statistics	Overall interpretation
Doherty et al. 2020	**Barriers to the implementation of clinical guidelines for maternal alcohol consumption in antenatal services: A survey using the theoretical domains framework**		
	Clinicians and managers face several challenges in implementing guidelines on maternal alcohol consumption	Lower mean scores in the survey indicate significant barriers. These barriers are different for clinicians and managers	Doctors, nurses and managers find it difficult to apply rules about drinking during pregnancy because of different challenges
	Environment and a lack of resources are major barriers for both groups	The ‘environmental context and resources’ domain scored low for both groups, indicating it as a major obstacle	The workplace environment and lack of necessary tools or resources are the biggest hurdles in applying these rules
	There is a need for comprehensive strategies addressing these barriers	The results suggest developing strategies that cater to both clinicians’ and managers’ challenges	When improving these guidelines, we need to address the challenges that both doctors/nurses and managers face to create effective strategies
Davies-Tuck, 2021	**Understanding staff views and experiences of a clinical practice change to reduce stillbirth in South Asian women: A cross-sectional survey** (A multicentre descriptive study was conducted involving 78 direct observations of provider-implemented protocols)		
	Most of the 120 surveyed staff agreed on the need for the guideline, its clarity and application. However, they reported increased workload and concerns around rationale, lack of education, resources, patient safety, access to care and communication issues with non-English speakers	Most staff understood the reasons behind the guidelines (79%), its intended audience (83%) and procedures (74%). Yet, 72% reported a workload increase and were concerned about the guideline’s various aspects	Most staff understood the reasons behind the guidelines (79%), its intended audience (83%) and procedures (74%). Yet, 72% reported a workload increase and were concerned about the guideline’s various aspects
Olmedo et al. 2014	**Improving maternal health and safety through adherence to postpartum haemorrhage protocol in Latin America**		
	There were three significant differences between the national, regional and local levels of care: adherence to all three interventions (P b 0.001); professional experience (P b 0.04) and retention of healthcare providers (P b 0.001). There were no differences in provider training (P b 0.097) and the retention of experienced healthcare providers was not associated with greater adherence to protocols Key barriers and opportunities for improving implementation were identified, especially regarding guidelines for culturally and linguistically diverse women	Adherence to AMTSL guidelines at National level 29% Regional level 3% Local level 46% This is a conclusion based on the staff’s responses. It suggests that while there are obstacles, there are also chances for improvement, particularly for guidelines targeting diverse populations	The present results highlight three important conclusions: physician training and experience did not affect protocol adherence; non-physician providers, who are more likely to practise at the lower levels of care, had better adherence to protocols; and institutional culture and beliefs seemed to have the greatest influence on healthcare professionals’ adherence to protocols The study found key obstacles and potential improvements in applying the guidelines, particularly when dealing with women from different cultural backgrounds and languages
Bell et al. 2018	**Implementation of national screening guidelines for gestational diabetes: A national survey of maternity units in England**		
	Health professionals from 113 (84%) of NHS Trusts in England responded to the survey. Most trusts (81%) offered OGTT at 26–28 weeks’ gestation to women with selected risk factors for GDM. However, almost 40% of trusts were not fully compliant with NICE screening criteria for all risk factors, mainly because of not offering OGTT to women with previous GDM (25% of trusts), BMI 30 kg/m^2^ or ethnic minority groups (22% of trusts). The main barriers to compliance with the BMI threshold were a lack of capacity, resources and funding given the high prevalence of maternal obesity. Forty-one per cent of trusts used diagnostic thresholds for GDM which differed from NICE recommendations	While most trusts are compliant with gestational diabetes screening protocols, 40% of trusts are not compliant	Maternity services in England are predominantly offering risk-factor-based screening for GDM. However, almost 40% of trusts do not fully comply with all the recommended risk factors for offering OGTT at 24–28 weeks gestation. Furthermore, nearly 40% of trusts do not comply with recommended diagnostic criteria, particularly, the amended fasting glucose threshold
Braddick, 2016	**A mixed- methods study of barriers and facilitators to the implementation of postpartum haemorrhage guidelines in Uganda**		
	The quantitative part of this study was to determine the level of adherence to postpartum haemorrhage clinical guideline recommendations Of 154 women, individual AMTSL, in the form of administering a uterotonic during the third stage of labour, controlled cord traction or delayed cord clamping, occurred in 105 (68.2%), 119 (77.3%) and of a subset of 60 patients, 37 (61.7%) individuals, respectively. However, only 18 of 53 (34.0%) individuals observed for receipt of all three AMTSL components received all the essential elements of AMTSL	Only 18 of 53 (34.0%) individuals observed for receipt of all the three AMTSL components received all the essential elements of AMTSL. This is a low implementation of PPH guidelines according to WHO	Overall guideline adherence was low
Murray-Davis et al. 2022	**Midwives’ perceptions of managing pregnancies complicated by obesity: A mixed methods study**		
	One hundred and forty-four midwives completed the survey and 20 participated in an interview. The participants described their clinical management when caring for those with obesity which included considerations regarding additional tests/investigations, consultation and transfer of care, and place of birth. Up to 93% of surveyed midwives believed that clients with obesity were appropriate for midwifery-led care; however, there was less certainty about suitability as BMI increased to higher ranges such as > 45	The majority of midwives reported awareness of obesity care protocols. They, however, had a difference in knowledge of when to transfer care whether below or above a BMI of 45 Most respondents reported that BMI guidelines were not well understood within the midwifery curriculum but through CPDs from professional organisations. The system influences of the use of guidelines such as collaborative care was generally split among the midwives with 50% agreeing with collaborative care while 50% disagreeing with the importance of collaborative care	Midwives in Ontario believe clients who are obese are suitable for midwifery-led care, but feel they have gaps in knowledge about the clinical implications of obesity and approaches to management. The lack of consistent guidelines and policies focussed on obesity in pregnancy has led to considerable variation among midwives and other care providers which has contributed to challenges for interprofessional collaboration. The participants articulated a desire to achieve a ‘healthy at every size’, individualised and non-judgemental approach underpinned by consistent clinical practice guidelines to inform clinical management
Kisakye et al. 2017	**Effect of support supervision on maternal and newborn health services and practices in rural Eastern Uganda**		
	There was a significant improvement in maternal and newborn services. For instance, across the first, second and third quarters, availability of parenteral oxytocin increased from 57% to 75% and to 82%. Removal of retained products of conception increased from 14% to 50% to 54% respectively There was a perceived improvement in the use of standards and guidelines for emergency obstetric care and quality of care	This mixed methods study showed improvements in maternal practice with support supervision. There was a steady improvement in performance in key maternal aspects in all the sites supervised	The use of multidisciplinary support supervision, mentorship and audit was a major contributor to the improvement

Note: Please see the full reference list of the article, Atsali EN, Kaura D, Tomlinson M. Effectiveness of dissemination strategies and use of maternal clinical guidelines: A narrative review. Afr J Prm Health Care Fam Med. 2024;16(1), a4494. https://doi.org/10.4102/phcfm.v16i1.4494 for more information.

CPD, continuous professional development; AMTSL, Active Management of Third Stage of Labour; NICE, National Institute for Healthcare Excellence; PPH, Post Partum Haemorrahge; WHO, World Health Organisation; OGTT, Oral Glucose Tolerance Test; GDM, Gestational Diabetis Mellitus; NHS, National Health Service..
